# Immunoreactivity of the 14F7 Mab Raised against N-Glycolyl GM3 Ganglioside in Primary Lymphoid Tumors and Lymph Node Metastasis

**DOI:** 10.1155/2013/920972

**Published:** 2013-11-28

**Authors:** Rancés Blanco, Damián Blanco, Yisel Quintana, Xiomara Escobar, Charles E. Rengifo, Marta Osorio, Zailí Gutiérrez, Janet Lamadrid, Mercedes Cedeño, Milagros Frómeta, Adriana Carr, Enrique Rengifo

**Affiliations:** ^1^Laboratory of Recognition and Biological Activity Assays, Department of Quality Control, Center of Molecular Immunology, P.O. Box 16040, 216 Street and 15 Avenue Atabey, Playa, 11600 Havana, Cuba; ^2^Department of Cell Biology and Tissues Banking, National Institute of Oncology and Radiobiology, 29 and F Street Vedado, Plaza de la Revolución, 10400 Havana, Cuba; ^3^Department of Pathology, Manuel Fajardo General Hospital, Zapata and D Street Vedado, Plaza de la Revolución, 10400 Havana, Cuba; ^4^Clinical Trials Unit, National Institute of Oncology and Radiobiology, 29 and F Street Vedado, Plaza de la Revolución, 10400 Havana, Cuba; ^5^Department of Pathology, National Institute of Oncology and Radiobiology, 29 and F Street Vedado, Plaza de la Revolución, 10400 Havana, Cuba; ^6^Research and Development Direction, Center of Molecular Immunology, P.O. Box 16040, 216 Street and 15 Avenue Atabey, Playa, 11600 Havana, Cuba

## Abstract

The reactivity of the 14F7 Mab, a highly specific IgG1 against N-glycolyl GM3 ganglioside (NeuGcGM3) in normal tissues, lymphomas, lymph node metastasis, and other metastatic sites was assessed by immunohistochemistry. In addition, the effect of chemical fixation on the 14F7 Mab staining using monolayers of P3X63Ag.653 cells was also evaluated. Moreover, the ability of 14F7 to bind NeuGcGM3 ganglioside inducing complement-independent cytotoxicity by a flow cytometry-based assay was measured. The 14F7 Mab was reactive in unfixed, 4% paraformaldehyde, 4% formaldehyde, and acetone fixed cells. Postfixation with acetone did not alter the localization of NeuGcGM3, while the staining with 14F7 Mab was significantly eliminated in both cells fixed and postfixed with methanol but only partially reduced with ethanol. The staining with 14F7 Mab was evidenced in the 89.2%, 89.4%, and 88.9% of lymphomas, lymph node metastasis, and other metastatic sites, respectively, but not in normal tissues. The treatment with 14F7 Mab affected both morphology and membrane integrity of P3X63Ag.653 cells. This cytotoxic activity was dose-dependent and ranged from 24.0 to 84.7% (10–1000 **μ**g/mL) as compared to the negative control. Our data could support the possible use of NeuGcGM3 as target for both active and passive immunotherapy against malignancies expressing this molecule.

## 1. Introduction

Lymphomas (primary malignant neoplasms of lymphoreticular origin) represent one of the major health problems worldwide [1]. Despite the availability of several options to the treatment of lymphomas [2], the identification of novel tumor-associated antigens that are universally expressed in these malignancies is necessary for the development of newer immunotherapeutic strategies [3].

On the other hand, as it is well known, the main cause of cancer-related death is due to metastasis of primary tumors to secondary sites within the body [4]. Usually, different primary tumors tend to spread to preferred metastatic sites, although both regional and distant lymph nodes are the most common metastatic targets for a variety of primary malignancies such as skin, breast, colon, stomach, and lung [5].

Gangliosides are sialic acid-containing glycosphingolipids engaged in many biological events that take place at vertebrate's cell membrane [6]. Unusual glycolylated gangliosides have been identified by immunohistochemical methods in some malignant tumors and their metastasis becoming attractive targets for immunotherapy [7–10].

The tissue reactivity of 14F7 Mab, a highly specific IgG1 against the NeuGcGM3 ganglioside, in a variety of frozen and formalin-fixed and paraffin-embedded malignant tumors have been previously published [8, 10–18]. Nevertheless, up to now, there is no evidence about the staining of 14F7 Mab in malignant lymphoma and nonmelanoma lymph node metastasis.

For these reasons, here we evaluated the reactivity of the 14F7 Mab in normal as well as in primary lymphoid tumors, lymph node, and other metastasis. Additionally, we assessed the effect of fixation in the recognition of 14F7 Mab as well as the ability of this Mab to bind NeuGcGM3 ganglioside inducing complement-independent cytotoxicity in a mouse myeloma cell line (P3X63Ag.653).

## 2. Materials and Methods

### 2.1. Cell Line and Monolayer Preparation

The mouse myeloma cell line P3X63Ag.653 (ATCC CRL-1580) was used. Cells were grown in Dulbecco's modified Eagle's media (PAA, E15-843) supplemented with 10% heat-inactivated fetal bovine serum (PAA, A15-211), respectively. Cells were maintained at 37°C in a humidified atmosphere of air containing 5% CO_2_ and the media was replaced every 3 to 4 days. Afterward, cultured cells were extensively washed with PBS (PAA, H15-002) and both cell viability and concentration were calculated by trypan blue exclusion assay followed by examination with a hemacytometer under an optical microscope. Only cells suspensions with viability greater than 90% were used. Then, cells were adjusted at 0.5 × 10^6^ cell/mL, deposed in histological slides, and air dried. Finally, slides were stored at −20°C until they were used.

### 2.2. Monoclonal Antibodies

We used the 14F7 Mab (IgG1), a highly specific anti-NeuGcGM3 ganglioside antibody. This Mab was generated by immunization of Balb/c mice with NeuGcGM3 hydrophobically conjugated with human very low-density lipoproteins (VLDL) adjuvated with Complete Freud adjuvant (CFA). Afterwards, 14F7 Mab was obtained by the hybridoma resulting of the fusion of spleen cells with mouse myeloma cell line P3X63Ag.653 as described [8]. Additionally, the P3 Mab (IgM, k, anti-NeuGc-containing gangliosides and sulfated glycolipids) [7] (complement-independent cytotoxicity assays) or the 1E10 Mab (an anti-idiotypic antibody specific for P3 Mab) [19] (immunocytochemistry assays) were used as negative controls.

### 2.3. Fixation Protocols and Immunocytochemical Procedure

Monolayers of P3X63Ag.653 cell line were fixed according to the following fixation protocols: 4% neutral buffered formaldehyde (Spectrum, F0110) and 4% paraformaldehyde (BDH, 294474L) for 30 and 20 minutes at room temperature, respectively, and acetone (Spectrum, A1020), ethanol (Spectrum, E1028), and methanol (Spectrum, M1240) for 10 minutes at 2–8°C each one. Unfixed cells were also included. Additionally, samples were prefixed with 4% neutral buffered formaldehyde before fixation with acetone, ethanol, and methanol fixatives.

Afterwards, cells were rinsed and rehydrated in tris saline buffer, pH 7.6 (TBS) for 10 minutes at room temperature. Then, samples were incubated for 1 h with 14F7 or 1E10 (irrelevant) antibodies. After two rinses, a labelled polymer-HRP (Dako, K1494) was added and the slides were incubated for 30 minutes. Finally, the enzymatic activity was visualized with AEC (Dako, K3461) and cells were contrasted with methyl green (Dako, S1962).

### 2.4. Tissue Specimens

Routinely processed, formalin-fixed, and paraffin-embedded archival samples with diagnosis of normal tissue (7), human lymphoma (37), lymph node metastasis of breast carcinoma (14), cutaneous malignant melanoma (17), colonic (12) and gastric (2) adenocarcinomas, lung cancer (1), and unknown primary tumor (1) as well as, other metastasis (9) were received from the pathology departments of both the National Institute of Oncology and Radiobiology and Manuel Fajardo General Hospital. All samples were used after the approved consent by the institutional ethical committees.

### 2.5. Previous Processing and Immunohistochemical Staining

Five microns serial sections from each block were obtained in a Lizt 1512 micrometer and mounted on plus slides (Dako, S2024). All sections were attached to the slide by heating in a 60°C oven for 1 hour. Afterwards, the slides were kept at room temperature and they were used within 30 days.

The slides were dewaxed in xylene and rehydrated in decreasing ethanol series as usually and endogenous peroxidase activity was blocked with dual endogenous enzyme block solution (Dako, S2003) for 10 minutes. Afterwards, all sections were washed in distilled water for 10 minutes and then were rinsed with TBS (Tris/saline buffer solution) for 5 minutes. The samples were incubated with the 14F7 Mabs for 1 hour at room temperature. Negative controls were performed by substituting primary antibody for TBS and sections of colonic adenocarcinoma of known positivity for NeuGcGM3 ganglioside were taken as positive control. After two rinses in TBS, the slides were incubated with a biotinylated link universal and streptavidin-HRP (Dako, K0690) for 30 minutes in each step. Between incubations, slides were washed with TBS for 10 minutes.

Afterwards, the enzymatic activity was visualized with a DAB (Dako, K3465) solution. For lymph node metastasis of malignant melanomas, AEC (Dako, K3461) was used. Finally, all slides were counterstained with Mayer's Hematoxylin (Dako, S2020), dehydrated, and mounted.

### 2.6. Immunostaining Evaluation

Staining of both cell membrane and cytoplasm was considered as positive for 14F7 Mab. For immunocytochemical assays, the intensity of reaction of each sample was judged as negative (−), weak (+), moderate (++), and strong (+++). For immunohistochemical determinations, a semiquantitative scoring system was used to define levels of reactivity. The recognition of 14F7 Mab was evaluated for percentage of positive cells (0–100%) and the intensity of reaction (0−3+). The score was calculated for each specimen by multiplication of the intensity of reaction and the grade of positive cells, resulting in a score ranging from 0 to 300. Subsequently, these scores were grouped as follows: 0 (score 0); 1 (scores 1–100); 2 (scores 101–200), and 3 (scores 201–300).

### 2.7. Complement-Independent Cytotoxicity

The induction of cell death procedures as previously described [20] with minor modifications was used. Briefly, P3X63Ag.653 cells were resuspended in culture medium with 1% bovine serum albumin (BSA) (Sigma, A2153) at 0.25 × 10^6^ cells/mL and incubated with 14F7 Mab (10–1000 *μ*g/mL) in a 5% CO_2_ atmosphere at 37°C for 2 hours. Cells were washed, re-suspended in FACSFlow (Becton-Dickinson, 342003) and maintained in ice bath. Afterwards, cells were resuspended with FACSFlow and stained with propidium iodide (PI) at a final concentration of 4 *μ*g/mL. The percentage of PI-positive cells was measured using a FACScan flow cytometer (Becton-Dickinson). Cells that were stained red with PI were considered as dead.

## 3. Results

### 3.1. The Recognition of 14F7 Mab Varies Depending on the Protocol Fixation

**Figure 1:**
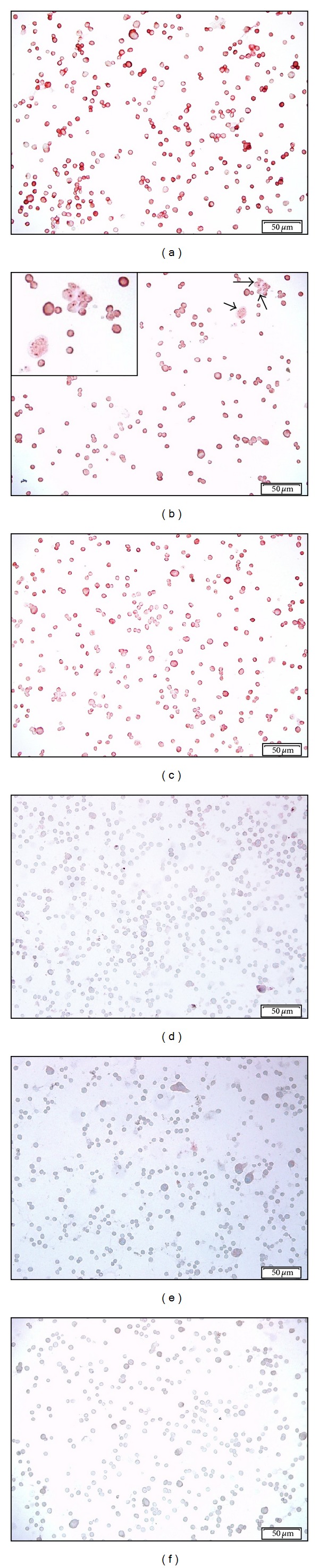
Microphotographs of the 14F7 Mab reactivity in P3X63Ag.653 cell line after different fixation protocols. An intense, homogeneous, and finely granular reactivity of 14F7 Mab was observed in cells fixed with 4% neutral-buffered formaldehyde (a). Observe: both the slight diminishing in the intensity of reaction and the appearance of cells with a grosser granular staining mainly located in the cytoplasm after fixation with acetone (b) (black arrows). Inset on the upper-left corner, 400x magnification. The 14F7 Mab staining was only partially diminished after fixation with ethanol (c) while, the reactivity with this Mab was significantly removed in cells fixed and postfixed with methanol (d and e, resp.). No immunoreaction was detected in cells incubated with 1E10 Mab (negative control) (d). White bar = 50 *μ*m.

The effect of a variety of fixation protocols on the 14F7 Mab immunoreaction was evaluated in monolayers of P3X63Ag.653 cell line (see Section 3). A homogeneous and finely granular reactivity of 14F7 Mab was evidenced in unfixed as well as in 4% neutral-buffered formaldehyde and 4% paraformaldehyde fixed cells (Table 1, Figure 1(a)). In acetone fixed cells, both a slight diminishing in the intensity of reaction with 14F7 Mab and the appearance of cells with a grosser granular staining mainly located in the cytoplasm were observed (Figure 1(b)). On the other hand, the staining with 14F7 Mab was only partially reduced after fixation (Figure 1(c)) and postfixation with ethanol while, postfixation with acetone did not alter the localization of the ganglioside (Table 2). The reactivity with 14F7 Mab was significantly eliminated in both cells fixed and postfixed with methanol (Figures 1(d) and 1(e)). No immunoreaction, independently of the fixation protocol, was detected in cells incubated with 1E10 Mab which was used as negative control (Figure 1(f)).

### 3.2. No Staining with 14F7 Mab Was Evidenced in Normal Tissues

No reactivity of 14F7 Mab was evidenced in normal tonsil (0/2), lymph node (0/2), and spleen (0/3).

### 3.3. Higher Levels of 14F7 Mab Reactivity Were Detected in Sections of Primary Lymphoid Tumors

**Figure 2:**
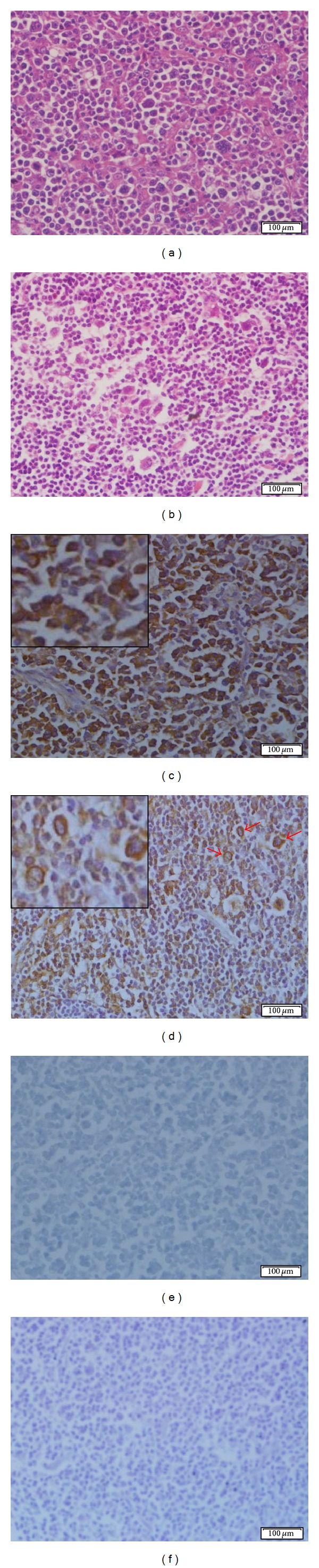
Microphotographs of the 14F7 Mab reactivity in human lymphoma. (a) and (b): Hematoxylin and eosin staining of diffuse large B-cell lymphoma and nodular sclerosis classical Hodgkin lymphoma, respectively. (c) and (d): Immunostaining with 14F7 Mab. Note: the intense reactivity of 14F7 Mab in both the cell membrane and cytoplasm of both malignant B lymphocytes (c) and Hodgkin cells (d) (red arrows). Inset on the upper-left corner, 400x magnification. (e) and (f): Negative controls. Observe: the absence of staining. White bar = 100 *μ*m.

The 14F7 Mab reactivity was detected in 33/37 (89.2%) of all studied lymphomas not depending on the histopathological subtype (Table 3). In 21/33 (63.6%) cases, a moderate to intense reaction with 14F7 Mab in more than 40% of malignant lymphocytes was observed, while 12/33 (36.4%) sections displayed a weak staining with this Mab. In general, the recognition of 14F7 Mab was observed as a finely granular reaction mainly located in the cell membrane but also in the cytoplasm of malignant cells (Figure 2). No immunostaining was evidenced in negative controls.

### 3.4. The 14F7 Mab Immunostaining Was Observed in Lymph Node Metastasis

**Figure 3:**
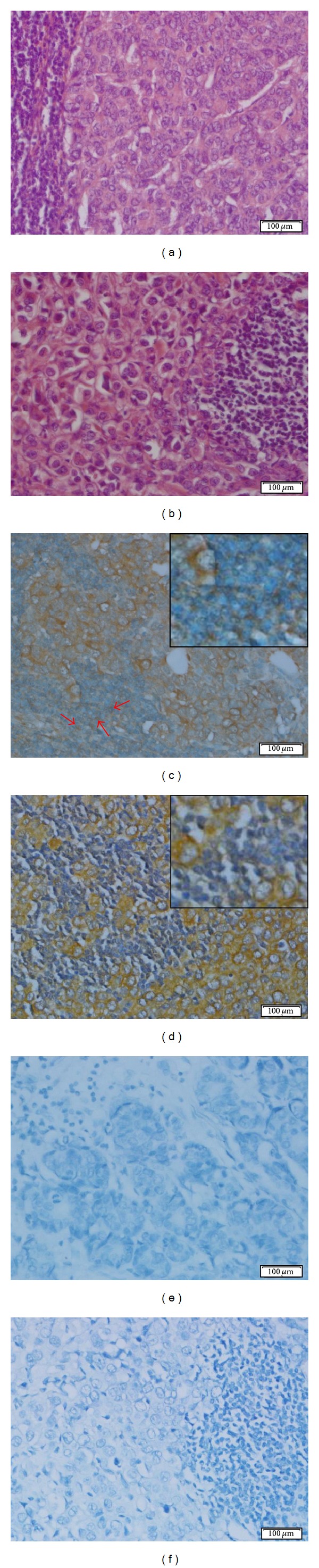
Microphotographs of the 14F7 Mab reaction in lymph node metastasis. (a) and (b): Hematoxylin and eosin staining of lymph node metastasis of breast ductal and lobular adenocarcinomas, respectively. (c) and (d): Immunostaining with 14F7 Mab. Observe: an intense reaction of 14F7 Mab located in both cell membrane and cytoplasm of malignant epithelial cells. See: an intense immunostaining in lymphocytes of tumor adjacent areas (c) (red arrows). Inset on the upper-right corner, 400x magnification. Note: the absence of reactivity in peritumoral lymphocytes (d). (e) and (f): Negative controls. Observe: the lack of staining. White bar = 100 *μ*m.

An intense reactivity of 14F7 Mab was detected in almost all tumor cells in 11/12 (91.7%) and 2/2 of lymph node metastasis of breast ductal and lobular carcinomas, respectively (Table 4). The recognition of 14F7 Mab was homogeneous and finely granular and was mainly located in the membrane of malignant cells although the cytoplasm was also decorated (Figure 3). Only one case of breast ductal carcinoma (8.3%) exhibited a weak to moderate and diffuse pattern of staining.

Concerning lymph node metastases of cutaneous melanoma, the reactivity of 14F7 Mab was detected in more than 50% of malignant cells in 15/17 (88.2%) (Table 4). The recognition of the 14F7 Mab was variable in the intensity of reaction (from weak to intense). A weak to moderate staining with 14F7 Mab was observed in 3/15 (20.0%) of samples while in 12/15 (80.0%) of them, a moderate to intense staining was evidenced. Only one case showed a moderate reaction with 14F7 Mab in less than 25% of malignant cells.

**Figure 4:**
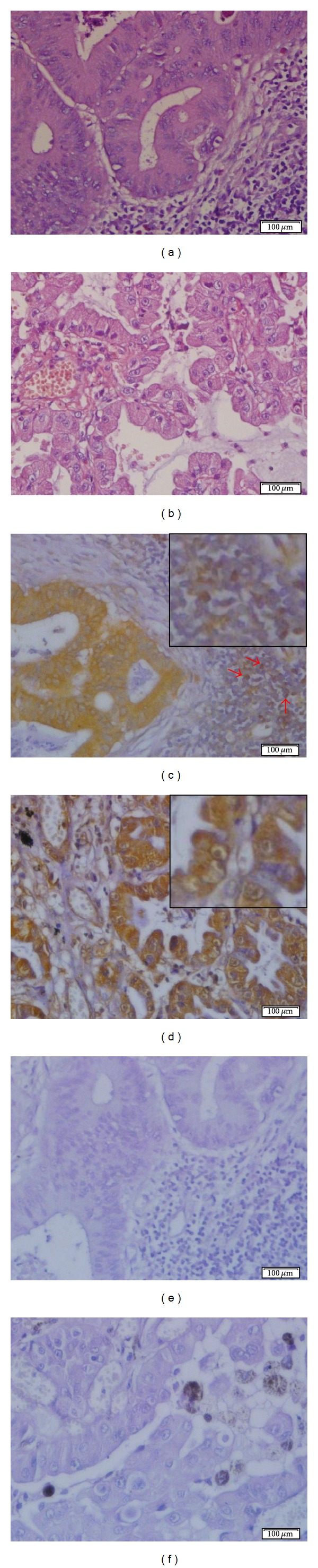
Microphotographs of the 14F7 Mab staining in lymph node metastasis. (a) and (b): Hematoxylin and eosin staining of lymph node metastasis of colonic and lung adenocarcinomas, respectively. (c) and (d): Immunoreactivity of the 14F7 Mab. Observe: an intense reaction of 14F7 Mab located in both cell membrane and cytoplasm of malignant epithelial cells. See: a moderate immunostaining in lymphocytes of tumor adjacent areas (red arrows). Inset on the upper-right corner, 400x magnification. (e) and (f): Negative controls. Observe: the lack of staining. White bar = 100 *μ*m.

Lymph node metastasis of colonic adenocarcinomas exhibited a homogeneous and finely granular reaction with 14F7 Mab in 9/12 (75.0%) cases, not taking into account the grade of differentiation (Table 4). The intensity of this reaction varied from moderate to intense and was observed in the cell membrane and also in the cytoplasm of more than 70% of malignant glandular cells (Figure 4). Similar results were evidenced in lymph node metastasis of gastric adenocarcinoma (2/2), lung adenocarcinoma (1/1) (Figure 4), and carcinoma of unknown primary origin (1/1).

Additionally, in 3/14 (21.4%) of lymph node metastasis of breast carcinoma as well as in 2/9 (22.2%) of colon adenocarcinomas, a weak to intense reaction of 14F7 Mab was observed in lymphocytes of tumor adjacent areas (Figures 3(a), 3(b), 4(a), and 4(b)).

### 3.5. Other Metastatic Sites Were Also Reactive with 14F7 Mab

A moderate to intense immunoreaction with 14F7 Mab was obtained in both cutaneous (1/1) and subcutaneous (3/3) metastasis of primary malignant melanoma. The staining was homogeneous and was mainly located on the plasmatic membrane of more than 90% of malignant cells although the cytoplasm was also reactive. Other metastatic tumors such as neuroblastoma (1/1), renal cell carcinoma (1/1), and those from unknown origin (2/2) were also recognized by 14F7 Mab. No reactivity was evidenced in a metastatic thymic carcinoma (Table 5).

### 3.6. Treatment with 14F7 Mab Induces a Complement-Independent Cytotoxicity in a Dose-Dependent Manner

**Figure 5:**
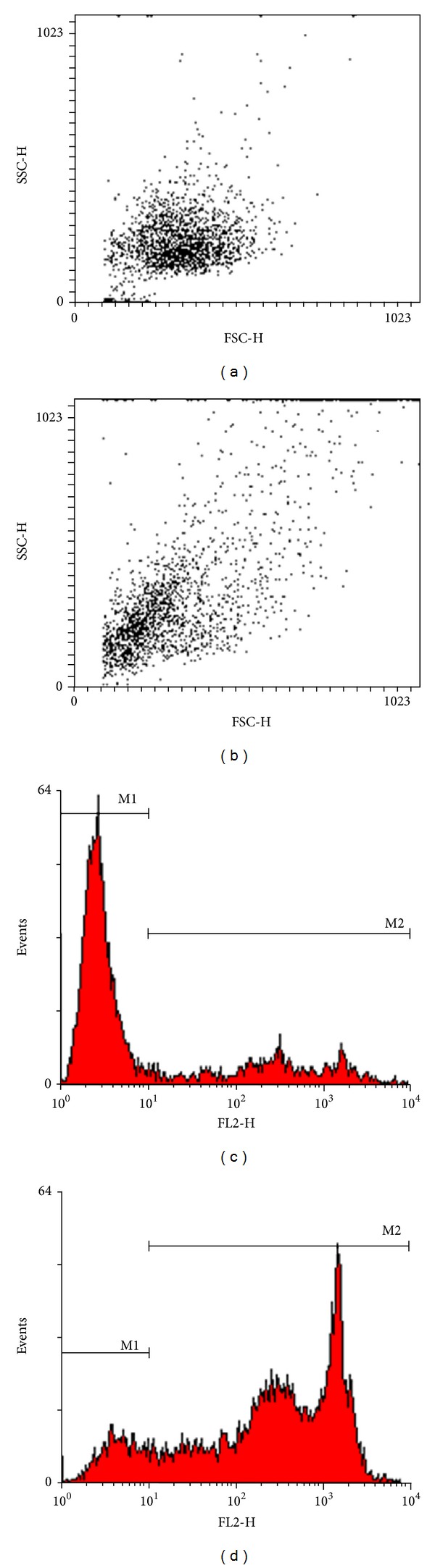
Representative flow cytometric profiles of complement-independent cytotoxicity of 14F7 Mab in the murine myeloma derived cell line, P3X63Ag.653. (a) and (b): Dot plots graphs. Correlated measurements of FSC-H (cell-surface area or size) and SSC-H (cell granularity or internal complexity) permit to detect changes in the light scattering properties of cells. Observe: a homogeneous population of cells treated with P3 Mab (irrelevant control) (a). The treatment with 14F7 Mab altered the morphology of P3X63Ag.653 cells, characterized by changes in cell size and granularity based on both FSC-H and SSC-H parameters analysis (b). (c) and (d): Histogram graphs showing the discrimination of viable (M1) and nonviable (M2) cells using the propidium iodide (PI) staining. These graphs correspond to (a) and (b), respectively. Nonviable cells uptake this dye fluorescing brightly in the red range of the visible color spectrum (FL2-H). A limited fraction of P3X63Ag.653 cells showed a positive staining with PI after incubation with P3 Mab (M2 region). Note: the dramatic decreasing in the cell viability after treatment with 14F7 Mab.

The ability of 14F7 Mab to induce a complement-independent cytotoxicity after binding to NeuGcGM3-positive cell line (P3X63Ag.653) was evaluated. This effect was tested by PI internalization and flow cytometry analysis. The treatment with 14F7 Mab affected both morphology and membrane integrity of tumor cells. After treating P3X63Ag.653 cells with 14F7 Mab, a subpopulation exhibiting both cell size (FSC-H) and internal complexity (SSC-H) alterations (Figures 5(a) and 5(b)), as well as a positive staining with PI were observed (Figures 5(c) and 5(d)). The cytotoxic activity of 14F7 Mab was dose-dependent and ranged from 24.0 to 84.7% as compared to the negative control, obtaining the maximum of cell death at 1000 *μ*g/mL. This effect was not seen when cells were treated with P3 Mab used as negative control.

## 4. Discussion

The use of frozen samples is desirable for any determination of gangliosides, but it is known, this kind of samples is often difficult to obtain and, even when available, to conserve. For these reasons, the most common specimen available for immunohistochemical assays after the histopathological diagnosis is formalin-fixed, ethanol and xylene-processed, and paraffin-embedded tissues. But, gangliosides are usually damaged or extracted after the routine treatment of tissues with organic solvents, due to their glycosphingolipid chemical structure.

In this paper, we demonstrated that cells fixation plays an important role for the immunolocalization of NeuGcGM3 by mean of 14F7 Mab. As expected, both fixation and postfixation with methanol completely removed NeuGcGM3 ganglioside from P3X63Ag.653 cells, consistent with the solubility of the glycolipid antigens [21]. While fixation with acetone only lead to slight decreasing in the intensity of reaction with 14F7 Mab joint to little alterations in the localization of NeuGcGM3. These results seem to be related to a complete partial extraction and/or displacing of this ganglioside from cells, similar to previous studies [21]. Consequently, methanol and acetone should be excluded as fixatives in the detection of NeuGcGM3 because both solubilize this ganglioside to different extents.

On the other hand, paraformaldehyde is one of the most common and adequate fixatives used in the immunolocalization of gangliosides [7, 8]. It is known that paraformaldehyde is a polymerization product of formaldehyde and to prevent its formation, formalin solutions usually contain different percentages of methanol (10–15%). Here, we obtained an intense reactivity of 14F7 Mab, mainly located in the membrane of P3X63Ag.653 cells after both 4% paraformaldehyde and 4% neutral-buffered formalin (NBF) fixations. These results permit to consider that the low percentage of methanol present in the final solution of NBF is not able to be removed NeuGcGM3 from cells. In this way, our data also could support the use of NBF fixation in the immunodetection of NeuGcGM3 ganglioside.

In addition, some authors have suggested that the routine tissues processing do not extract or damage the antigenic carbohydrate determinants of gangliosides [22]. Curiously, fixation and postfixation with ethanol only induce a partial extraction and/or damage of NeuGcGM3 ganglioside from the P3X63Ag.653 myeloma cells. These results are suggestive that carbohydrate determinant recognized by 14F7 probably resists the formalin fixation and the ethanol tissues processing, permitting its detection in this kind of samples. In line with this, the expression of NeuGcGM3 and NeuGcGD1a gangliosides in formalin-fixed and paraffin-embedded tissues using GMR8 Mab by immunohistochemical and TLC immunostaining methods has been recently demonstrated [23].

Aberrant and elevated expression of some gangliosides has been previously demonstrated on the surface of lymphoid derived tumors [24, 25]. In previous studies, GM3 was the predominant ganglioside found in pre-B lymphoma cells and B-cell neoplasms using over-pressured thin-layer chromatography (OPTLC) followed by scanning densitometry and high performance thin layer chromatography (HPTLC) and immune thin layer chromatography (ITLC), respectively. Nevertheless, these studies have been restricted to gangliosides containing the N-acetylated variant of sialic acid (NeuAc) [24, 25].

In the present work, we reported for the first time the tissue reactivity of the 14F7 Mab, a highly specific IgG1 against the N-glycolylated variant of GM3 ganglioside, in a variety of lymphoid tumors not related with the histopathological typing, but not in normal tissues. The pattern of staining of 14F7 was finely granular, homogeneous, and mainly located in cell membrane of malignant lymphocytes, similar to that observed in preceding reports in other tumor localizations [10, 14–17].

The expression of the N-glycolylated variant of sialic acid (NeuGc) forming the structure of gangliosides and/or other glycoconjugates (Hangnutziu-Deicher antigen, HD) in humans has been considered as a tumor-associated antigen [26]. Normal cells are capable to synthetize the sialic acid N-acetylneuraminic acid but not N-glycolylneuraminic acid (as a result of a deletion in the cytidine monophospho-N-acetylneuraminic acid hydroxylase gene). The aberrant expression of NeuGc residues in humans has been related to the metabolic incorporation from red meat and dairy products in the diet due to the altered metabolism of malignant cells [27–29]. However, some authors have reported an alternative pathway to the NeuGc synthesis from other intermediates of cellular metabolism in some human tumors [27]. The expression of NeuGc also triggers the immune responses to the nonhuman sialic acid, which is the mechanism of the “serum sickness” reaction previously described as HD antibodies [30, 31]. These HD antibodies were found to react with NeuGc bound to horse serum glycoconjugates and were then discovered in the serum and tissues of patients with cancer [30] and in activated and malignant immune cells [32].

The expression of heterophile HD antigen on the cell surface of both leukemia and malignant lymphoma was evidenced by membrane immunofluorescence staining with chicken antiserum against NeuGcGM3 and fluorescein-conjugated rabbit anti-chicken IgG [26]. The aberrant expression of NeuGc forming part of human lymphoma glycosphingolipids using gas chromatography-mass spectrometry had been also demonstrated [33]. Moreover, the expression of HD antigen in both pathologic sera and extracts of cancer tissues from lymphoma and leukemia had been previously detected by means of inhibition of agglutination of bovine erythrocytes by HD antibodies [30]. However, in other studies, no serum antibodies against HD antigen were detected in patients bearing lymphomas [34].

Additionally, we found the recognition of 14F7 Mab in most of lymph node metastasis of some primary tumors such as colonic and gastric adenocarcinoma, cutaneous malignant melanoma, breast tumors, and lung carcinoma. A preliminary study of the 14F7 Mab staining in cutaneous and subcutaneous metastasis of melanoma and other metastasis was also showed. Similar pattern of immunostaining and percentages of positive cells were obtained by our group in the corresponding primary malignancies [10, 14–16]. In previous reports, the 14F7 Mab was able to intensely stain cells of metastatic lymph nodes of cutaneous malignant melanoma [9, 14]. Moreover, another Mab (P3), reported by our group, binds to several NeuGc containing gangliosides and also reacts with sulfated glycolipids showed a positive staining in lymph node metastasis of breast tumors [7]. Interestingly, specific therapies anti-NeuGcGM3 ganglioside using molecular cancer vaccines have shown promising results in patients bearing lymph node metastasis of breast tumors and cutaneous malignant melanoma [35–37]. In this way, although in some cases the number of metastasis samples is small, it would be interesting to extent the evaluation of NeuGcGM3 expression in these malignancies in order to assess its potential use as target for immunotherapy.

On the other hand, gangliosides can be actively shed from the cell surface of malignant cells and taken up by other cells by insertion of their lipid anchors into the membrane [38]. In addition, shedding of immunosuppressive gangliosides to the tumor microenvironment has been reported to be an important characteristic of human malignancies [39]. These circulating gangliosides are potentially involved in tumor-host interactions contributing to cancer progression [40] and metastasis through tumor immune escape [41].

Here, it was observed a reactivity of 14F7 Mab in the cell membrane of normal lymphocytes localized around both metastatic breast carcinoma cells of axillary lymph nodes and metastatic colon adenocarcinoma cells of local lymph nodes (Figures 3(b) and 4(b), red arrows). It is known that sentinel lymph nodes are the first nodes to receive lymphatic drainage from a primary tumor and the preferential site of initial tumor metastasis. These lymph nodes are intensively exposed to the bioactive products of tumor cells and other associated cells [42]. Increased tumor-derived NeuGcGM3 ganglioside in sentinel lymph nodes have been suggested to directly influence coordinated interactions between DC and both helper and regulatory CD4^+^ T cells abrogating an anti-tumor-specific immune response [41]. In this way, our results seem to be in agreement with the potential shedding of NeuGcGM3 ganglioside by metastatic epithelial cells in secondary lymphoid organs as well as with its incorporation to the cell membrane of peritumoral lymphocytes.

Finally, we showed the ability of 14F7 murine Mab to directly kill the NeuGcGM3-positive murine myeloma cells without participation of complement. This Mab, after binding to NeuGcGM3, significantly induced the appearance of a subpopulation with increased cell size and internal complexity measured by flow cytometry. In addition, a decreasing in the tumor cell viability in a dose-dependent manner using the staining with PI was also observed in this subpopulation. Similar results had been previously reported [20, 43]. Nevertheless, the selection of patients to any anti-NeuGcGM3 specific therapies has been usually based on the tissue reactivity of the 14F7 murine Mab, which is typically used only for diagnostic purposes. It is known that therapy with murine-derived Mabs is limited by their tendency to develop human anti-mouse antibodies response (HAMA), the relatively short half-life of the Mabs and the limited ability of murine Fc to activate human immune mechanisms [44]. For that reason, in the last few years, a chimeric and humanized variant of 14F7 Mab were developed in our center. The resulting recombinant Mabs exhibited a similar ability to bind NeuGcGM3 and to induce complement- independent cytotoxicity as compared with the murine counterpart [20, 45]. In this sense, 14F7 Mab becomes a useful tool to identify tumors that could be sensitive to anti-NeuGcGM3 specific therapies, including passive immunotherapy using a chimeric and/or the humanized version of this Mab.

## 5. Conclusions

In summary, we reported the tissue reactivity of 14F7 Mab in both primary and secondary malignancies of lymphoid system. Evidence that probably support the potential resistance of the antigenic determinant recognized by 14F7 to formalin fixation and tissues processing were also showed. In addition, the capacity of 14F7 Mab to induce complement-independent cytotoxicity in a myeloma-derived cell line after binding NeuGcGM3 was demonstrated. Our data could support the possible use of NeuGcGM3 as target for both active and passive immunotherapy of malignancies expressing this molecule. Moreover, our results permit to consider the use of 14F7 murine Mab to identify lymphoid tumors that could be sensitive to anti-NeuGcGM3 specific therapies. Experiments in order to evaluate the recognition of 14F7 Mab in human leukemia are ongoing by our group.

## Figures and Tables

**Table 1:** Effect of different fixatives in the 14F7 Mab reactivity.

Intensity of reaction	Unfixed	NBF	PFA	Acetone	Ethanol	Methanol
	+++	+++	+++	++	++	−

Legend: NBF: neutral buffered formalin; PFA: paraformaldehyde. Intensity: − negative, ++ moderate, +++ intense.

**Table 2:** Effect of postfixation in the 14F7 Mab recognition.

Intensity of reaction	NBF	NBF/acetone	NBF/ethanol	NBF/methanol
	+++	+++	++	−

Legend: NBF: neutral buffered formalin. Intensity: − negative, ++ moderate, +++ intense.

**Table 3:** Immunostaining of 14F7 Mab in primary lymphoid tumors.

Lymphoid tumors	Score				
	0	1	2	3	Total (%)
Mature B-cell neoplasms					
Small lymphocytic lymphoma	0	2	1	1	4/4
Plasma cell myeloma	0	0	1	1	2/2
Follicular lymphoma	0	3	1	0	4/4
Diffuse large B-cell lymphoma	2	4	6	6	16/18 (88.9)
Burkitt lymphoma	0	1	0	0	1/1
Classical Hodgkin lymphoma	1	1	1	0	2/3 (66.7)
Unclassified	1	0	1	3	4/5 (80.0)

Legend: 0 (score 0); 1 (scores 1–100); 2 (scores 101–200), and 3 (scores 201–300).

**Table 4:** Immunorecognition of 14F7 Mab in some lymph node metastasis.

Lymph node metastasis of	Score				
	0	1	2	3	Total (%)
Cutaneous melanoma	2	2	8	5	15/17 (88.2)
Breast carcinoma					
Ductal	0	0	0	12	12/12
Lobular	0	0	0	2	2/2
Peritumoral lymphocytes	11	1	1	1	3/14 (21.4)
Colonic adenocarcinoma	3	0	3	6	9/12 (75.0)
Peritumoral lymphocytes	7	1	1	0	2/9 (22.2)
Gastric adenocarcinoma	0	0	1	1	2/2
Lung carcinoma	0	0	0	1	1/1
Unknown primary origin	0	0	1	0	1/1

Legend: 0 (score 0); 1 (scores 1–100); 2 (scores 101–200), and 3 (scores 201–300).

**Table 5:** Immunoreactivity of 14F7 Mab in other metastasis.

Metastasis of	Score				
	0	1	2	3	Total (%)
Cutaneous melanoma					
Subcutaneous	0	0	1	2	3/3
Cutaneous	0	0	0	1	1/1
Thymic carcinoma	1	0	0	0	0/1
Neuroblastoma	0	0	0	1	1/1
Renal cell carcinoma	0	0	0	1	1/1
Unknown primary origin	0	1	0	1	2/2

Legend: 0 (score 0); 1 (scores 1–100); 2 (scores 101–200), and 3 (scores 201–300).
